# Virulence determinants and multidrug resistance of emerging *Salmonella enterica* subspecies *salamae* in broiler chickens: a potential zoonotic concern

**DOI:** 10.1007/s11259-026-11163-x

**Published:** 2026-04-17

**Authors:** Iman Shaheed, Ahmed Samir, Elshaimaa Ismael, Ahmed Orabi, Sabah Ali, Basma Mohamed, Hazem Darwish, Fatma Abdel-Kader, Hala Zaher

**Affiliations:** 1 Department of Pathology, Faculty of Veterinary Medicine, Cairo University, Cairo, Egypt; 2 Department of Microbiology, Faculty of Veterinary Medicine, Cairo University, Cairo, Egypt; 3 Department of Veterinary Hygiene and Management, Faculty of Veterinary Medicine, Cairo University, Cairo, Egypt; 4 Technical and Marketing Manager, MSD Animal Health, Cairo, Egypt; 5 Department of Zoonoses, Faculty of Veterinary Medicine, Cairo University, Cairo, Egypt

**Keywords:** *Salmonella enterica*, Multidrug resistance, Virulence, Broilers, Zoonoses

## Abstract

*Salmonella* is a relevant zoonotic food-borne pathogen worldwide. Consequently, a significant amount of research has been focused on *Salmonella enterica* serovars over a substantial period of time. By far, investigating non-*enterica* subspecies in food-producing animals is an overlooked issue. Therefore, this study aimed to examine the prevalence, antimicrobial resistance, and virulence determinants of emerging *Salmonella* subspecies in broiler chickens in Egypt. Overall, salmonellae were identified in 19 out of 240 broiler chickens. This study revealed an unexpected distribution of serovars, with *S. enterica* subsp. *salamae* being identified in 31.6% of broilers, trailing behind *S*. Enteritidis (63.2%) and followed by *S*. Infantis (5.3%). Multidrug resistance accounted for 73.7% of the isolates, with four isolates of *S. enterica* subsp. *salamae* exhibiting a multiple antibiotic resistance index (MARI) greater than 0.2. A total of 19 antibiotic resistance and 7 virulence genes were investigated among 19 *Salmonella* isolates, showing varying frequencies. All six *S. enterica* subsp. *salamae* strains carried *tetA*, *int1*, and *bla*_TEM_, as well as six virulence genes (*stn*, *spiA*, *sopB*, *ompF*, *ompA*, and *pefA*), with *spvB* detected in five. Furthermore, a phylogenetic analysis of the *Salmonella* enterotoxin (*stn*) gene from two *S. enterica* subsp. *salamae* isolates revealed genetic similarities to isolates identified in wild birds and humans, highlighting their zoonotic potential. These outcomes shed light on the emerging MDR and virulent *S. enterica* subsp. *salamae*, which have raised a potential public health threat and necessitate strict monitoring and surveillance of non-*enterica* subspecies in the poultry sector.

## Introduction

Salmonellosis stands out as a major cause of foodborne illnesses worldwide, significantly affecting both human and animal health (Lamichhane et al. 2024). It is commonly transmitted to humans through food sources, posing a substantial public health threat (Teklemariam et al. 2023). Poultry, in particular, is a major contributor to salmonellosis outbreaks (Ammar et al. 2019), accounting for approximately 40% of clinically reported cases (Shalaby et al. 2022). Salmonellosis in humans typically manifests through acute fever, abdominal pain, diarrhoea, nausea, and vomiting. However, individuals with weakened immune systems, children under the age of five, and elderly people are at higher risk of experiencing more severe symptoms (Teklemariam et al. 2023). The genus *Salmonella* is categorized into two species: *Salmonella enterica* and *Salmonella bongori*. *S. enterica* is a prominent foodborne pathogen, commonly associated with human gastroenteritis. This species is further divided into six subspecies: *enterica* (subsp. I), *salamae* (subsp. II), *arizonae* (subsp. IIIa), *diarizonae* (subsp. IIIb), *houtenae* (subsp. IV), and *indica* (subsp. VI) (Yang et al. 2025). *S. enterica* has garnered significant interest from researchers, though this attention has been unevenly distributed, with a primary focus on *S. enterica* subsp. *enterica* (Lamas et al. 2018). Currently, *Salmonella* subspecies *enterica* represents a highly diverse species, encompassing more than 2,600 serovars owing to the significant variation in lipopolysaccharide (O) antigens and flagellar protein (H) antigens (Han et al. 2024). Among these, the serovars Typhimurium and Enteritidis have been recognized as the most prevalent serotypes found in Egyptian poultry farms (El Mansy et al. 2025).

The indiscriminate and widespread use of antimicrobial agents in the veterinary sector has significantly contributed to the development and dissemination of antimicrobial resistance globally (Samir et al. 2024, 2025; Shaheed et al. 2025; Shaker et al. 2025). This issue has been exacerbated by extensive antibiotic usage in both humans and animals, leading to an alarming rise in multidrug-resistant (MDR) *Salmonella*, as highlighted in recent reports (Musa et al. 2024; Fahmy et al. 2025). Taken into account, both antimicrobial resistance and virulence-associated genes play a crucial role in determining the virulence potential of pathogenic bacteria (Wetchasirigul et al. 2025). Notably, a wide array of virulence genes are implicated in the pathogenesis of salmonellosis, including the colonization of host tissues, the survival and replication of the pathogen within host cells, the invasion of phagocytic and non-phagocytic cells, and the dissemination of resistance among various strains (Zhao et al. 2025). Data on antimicrobial resistance and virulence in non-*enterica* subspecies remain limited, primarily due to their lower pathogenicity compared to *enterica* subspecies. Additionally, non-*enterica* subspecies have been less frequently isolated than the serotypes belonging to subspecies *enterica* (Lamas et al. 2018). In Egypt, numerous studies have examined the prevalence, antimicrobial resistance, and virulence of *S. enterica* subsp. *enterica* serovars in broiler chickens (Yu et al. 2021; Shalaby et al. 2022; Tohamy et al. 2022; Algammal et al. 2023). Yet, there is a significant gap in the prevalence of non-*enterica* subspecies in the broiler sector. As a result, this study seeks to address that knowledge gap by focusing on the emerging *Salmonella* subspecies, alongside investigating their virulence genes as well as the phenotypic and genotypic profiles of antimicrobial resistance in broiler chickens in Egypt.

## Materials and methods

### Study design and sample size

This study was conducted on 22 broiler farms between February 2023 and June 2023 across eight governorates in Lower Egypt (Sharqia, Qalyubia, Gharbia, and Beheira) and Middle Egypt (Giza, Fayoum, Bani Sweif, and Minya) to investigate the association between various factors and the prevalence of *Salmonella* infection in broiler chickens. The sample size was determined using the “Sample Size Calculator for Prevalence Studies”, relying on prevalence data of *Salmonella* spp. reported in previous studies conducted on broilers in Egypt. These studies documented prevalence rates of 14% (Nabil et al. 2022), 15.6% (Ammar et al. 2019), and 16.66% (Ibrahim et al. 2013). The required sample size was calculated using a 95% confidence level and a 5% margin of error, following the formula N = *Z*^*2*^ *P*(1 − *P*)/*d*^*2*^. Therefore, this study included 240 broiler chickens. Broiler flocks included in this survey ranged from 3 to 35 days of age, and age categories were summarized in weeks for statistical comparison. The sampled flocks represented common commercial genetic lines (Ross, Cobb, Ivan-48, and Sasso). The birds were humanely slaughtered by competent personnel through severing both jugular veins, both carotid arteries, and the trachea at the neck using a sharp knife, to ensure rapid loss of consciousness and effective exsanguination, following the American Veterinary Medical Association (AVMA) Guidelines for the Euthanasia of Animals (AVMA, 2021). Internal organs (liver and heart) were collected under aseptic conditions, placed into sterile, labelled sterile cups, and transferred within one hour in an icebox at 4 °C for bacteriological analysis. Additionally, data on potential factors were gathered through structured questionnaires and direct observations, including farm location (governorate and region), management practices (housing type, ventilation system, and bird density), flock characteristics (age and breed), and antibiotic usage.

### Isolation and identification of *Salmonella*

The heart and liver of each individual bird were pooled to create one sample per bird. *Salmonella* spp. were isolated according to the guidelines of the International Organization for Standardization (ISO) ISO 6579-1:2017 (ISO 2017). A 25 g portion of the homogenized sample was mixed with 225 mL of buffered peptone water (BPW) and incubated at 37 °C for 24 h. From each pre-enriched homogenate, 1 mL was aseptically transferred into 10 mL of Rappaport-Vassiliadis (RV) broth and further incubated at 42 °C for 24 h. Subsequently, the broths were streaked onto Xylose-Lysine-Desoxycholate (XLD) agar and incubated at 37 °C for 24 h. Colonies appearing as pure pink with a black center on XLD agar were considered presumptive *Salmonella* spp. Following the method described by Ayichew et al. (2024), these colonies were biochemically confirmed as *Salmonella* using various tests, including urea hydrolysis, H_2_S production on triple sugar iron agar, lysine decarboxylation, the indole test, the methyl red test, the Voges-Proskauer test, and the citrate utilization test.

### Serological identification of *Salmonella* isolates

The serotyping of suspected *Salmonella* strains was performed at the Animal Health Research Institute in Dokki, Giza, Egypt, following the guidelines provided by the manufacturer (Denka Seiken Co., Tokyo, Japan). The process was conducted on *Salmonella* isolates based on the Kauffman–White Scheme, which involves identifying somatic (O) and flagellar (H) antigens using both polyvalent and monovalent antisera (Jønsson et al. 2025).

### Antimicrobial susceptibility testing of obtained *Salmonella* isolates

Antimicrobial susceptibility testing (AST) was carried out on 19 *Salmonella* isolates using the Kirby-Bauer disc diffusion method, following the guidelines set by the Clinical and Laboratory Standards Institute (CLSI) (CLSI 2025). The isolates were tested against a panel of 21 antimicrobial agents, which included: ampicillin (10 µg), cefotaxime (30 µg), ceftazidime (30 µg), cefpodoxime (10 µg), cefoxitin (30 µg), cefepime (30 µg), cefoperazone (75 µg), aztreonam (30 µg), ertapenem (10 µg), meropenem (10 µg), fosfomycin (200 µg), gentamicin (10 µg), amikacin (30 µg), azithromycin (15 µg), tetracycline (30 µg), doxycycline (30 µg), ciprofloxacin (5 µg), nalidixic acid (30 µg), trimethoprim-sulfamethoxazole (1.25/23.75 µg), chloramphenicol (30 µg), and nitrofurantoin (300 µg). The diameter of the inhibition zone surrounding the discs was measured and evaluated against CLSI breakpoints. Isolates showing resistance to three or more antimicrobial classes were defined as multidrug-resistant (MDR) (Magiorakos et al. 2012). In addition, the multiple antibiotic resistance index (MARI) was calculated for MDR *Salmonella* isolates using the equation a/b, where “a” represents the number of antimicrobials to which an isolate was resistant, and “b” denotes the total number of antimicrobials tested (Singh et al. 2025).

### Genomic DNA extraction

DNA from bacterial pure culture was extracted using the conventional boiling method, as described by Siddiky et al. (2021). Briefly, each isolate was first grown on nutrient agar and incubated overnight at 37 °C. Fresh colonies from the overnight culture were harvested and suspended in nuclease-free water. The bacterial suspension was then boiled at 95 °C for 15 min and immediately cooled on ice. Finally, cell debris was separated by centrifugation, and the resulting supernatant containing the DNA template was stored at -20 °C for further use.

### Molecular detection of antimicrobial resistance genes

*Salmonella* isolates were subjected to polymerase chain reaction (PCR) to identify various antimicrobial resistance genes, including tetracycline resistance genes (*tetA* and *tetB*), sulfonamide resistance genes (*sul1* and *sul2*), the macrolide resistance gene (*ermB*), class 1 integron (*intI1*), quinolone resistance genes (*qnrA*, *qnrB*, and *qnrS*), the florfenicol resistance gene (*floR*), β-lactamase genes (*bla*_TEM_, *bla*_SHV_, *bla*_OXA_, *bla*_CTX−M,_ and *bla*_CYM−2_), and carbapenemase genes (*bla*_KPC_, *bla*_NDM_, *bla*_OXA−48_, and *bla*_VIM_). PCR assays were adjusted in a 25 µL reaction mixture consisting of 3 µL of DNA template (50–100 ng/µL), 12.5 µL of Cosmo PCR RED Master Mix (Willowfort, UK), 1 µL of each forward and reverse primer (10 pmol/µL), and 8.5 µL of nuclease-free water. Details of the primers used to target specific antimicrobial resistance genes are outlined in Table 1.

**Table 1:** Oligonucleotide primer sequences employed for PCR detection of antibiotic resistance genes in *Salmonella* spp

Target gene	Primer sequence (5ʹ–3ʹ)	Amplicon size (bp)	Reference
*tetA*	GGCCTCAATTTCCTGACG AAGCAGGATGTAGCCTGTGC	372	(Iweriebor et al. 2015)
*tetB*	CCTCAGCTTCTCAACGCGTG GCACCTTGCTCATGACTCTT	634	(Sadiq et al. 2017)
*sul1*	CGCACCGGAAACATCGCTGCAC TGAAGTTCCGCCGCAAGGCTCG	163	(Suhartono, 2016)
*sul2*	TCCGGTGGAGGCCGGTATCTGG CGGGAATGCCATCTGCCTTGAG	191	(Suhartono and Savin 2016)
*ermB*	GATACCGTTTACGAAATTGG GAATCGAGACTTGAGTGTGC	364	(Ateba et al. 2020)
*Int1*	CCTCCCGCACGATGATC TCCACGCATCGTCAGGC	280	(Sun et al. 2022)
*qnrA*	TCAGCAAGAGGATTTCTCA GGCAGCACTATGACTCCCA	627	(Kehrenberg et al. 2006)
*qnrB*	TCGGCTGTCAGTTCTATGATCG TCCATGAGCAACGATGCCT	469	(Kehrenberg et al. 2006)
*qnrS*	GACGTGCTAACTTGCGTGAT TGGCATTGTTGGAAACTTG	118	(Di Cesare et al. 2015)
*floR*	CACGTTGAGCCTCTATAT ATGCAGAAGTAGAACGCG	868	(El-Tayeb et al. 2017)
*bla*_TEM_	CGC CGC ATA CAC TAT TCT CAG AAT GA ACG CTC ACC GGC TCC AGA TTT AT	445	(Samir et al. 2024)
*bla*_SHV_	CTT TAT CGG CCC TCA CTCAA AGG TGC TCA TCA TGG GAA AG	237	(Samir et al. 2024)
*bla*_CTX−M_	ATG TGC AGY ACC AGTAAR GTK ATG GC TGG GTR AAR TAR GTS ACC AGA AYC AGC GG	593	(Samir et al. 2024)
*bla*_OXA_	ACA CAA TAC ATA TCA ACT TCG C AGT GTG TTT AGA ATG GTG ATC	813	(Samir et al. 2024)
*bla*_CYM−2_	AGCGATCCGGTCACGAAATA CCCGTTTTATG CACCCATGA	695	(Samir et al. 2024)
*bla*_KPC_	ATG TCA CTG TAT CGC CGT CT TTT TCA GAG CCT TAC TGC CC	882	(Li et al. 2012)
*bla*_NDM_	GGT TTG GCG ATC TGG TTT TC CGG AAT GGC TCA TCA CGA TC	621	(Li et al. 2012)
*bla*_OXA−48_	TTG GTG GCA TCG ATT ATC GG GAG CAC TTC TTT TGT GAT GGC	743	(Li et al. 2012)
*bla*_VIM_	GGT CTC ATT GTC CGT GAT GGT GAT GAG CTC GAT GAG AGT CCT TCT AGA G	271	(Li et al. 2012)

### Molecular investigation of *Salmonella* virulence genes

The identification of *Salmonella* virulence factors was conducted using uniplex PCR, targeting gene sequences such as *stn*, *spvB*, *spiA*, *sopB*, *ompF*, *ompA*, and *pefA*, as listed in Table 2. Each PCR reaction was performed in a final volume of 25 µl, containing 12.5 µl of Cosmo PCR RED Master Mix (Willowfort, UK), 1 µl of each primer at a concentration of 10 pmol, 4.5 µl of nuclease-free water, and 3 µl of DNA template (50–100 ng/µL). The resulting PCR products, representing antibiotic resistance and virulence-associated genes, were analyzed through gel electrophoresis on a 1.5% agarose gel. The gels were subsequently stained using ethidium bromide (Sigma-Aldrich, USA), visualized under ultraviolet light, and documented using a gel imaging system (BioRad, USA).

**Table 2:** Oligonucleotide primer sequences used for PCR detection of virulence genes in *Salmonella* spp

Target gene	Primer sequence (5ʹ–3ʹ)	Amplicon size (bp)	Reference
*pefA*	GCGCCGCTCAGCCGAACCAG GCAGCAGAAGCCCAGGAAACAGTG	157	(Lozano-Villegas et al. 2023)
*spvB*	CTATCAGCCCCGCACGGAGAGCAGTTTTTA GGAGGAGGCGGTGGCGGTGGCATCATA	717	(Lozano-Villegas et al. 2023)
*spiA*	CCAGGGGTCGTTAGTGTATTGCGTGAGATG CGCGTAACAAAGAACCCGTAGTGATGGATT	550	(Lozano-Villegas et al. 2023)
*sopB*	CGGACCGGCCAGCAACAAAACAAGAAGAAG TAGTGATGCCCGTTATGCGTGAGTGTATT	220	(Lozano-Villegas et al. 2023)
*stn*	TTG TGT CGC TAT CAC TGG CAA CC ATT CGT AAC CCG CTC TCG TCC	617	(Ghoneim et al. 2017)
*ompA*	AGT CGA GCT CAT GAA AAAGAC AGC TAT CGC AGT CAA GCT TTT AAG CCT GCG GCT GAG TTA	1052	(Kataria et al. 2013)
*ompF*	CCTGGCAGCGGTGATCC TGGTGTAACCTACGCCATC	519	(Tatavarthy and Cannons 2010)

### Partial sequencing of the *S. enterica* subsp. *salamae stn* gene and phylogenetic analysis

**Fig. 1:**
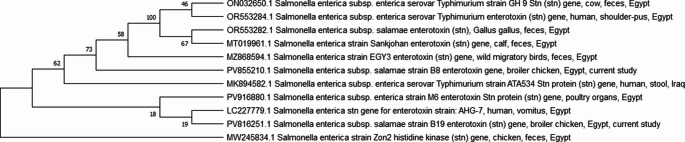
A phylogenetic bootstrap consensus tree was inferred via a maximum-likelihood method using MEGA 7 software to show the genetic relatedness between partial *S. enterica* subsp. *salamae stn* gene sequences retrieved in this study and *Salmonella* strains available on the GenBank database

Two MDR *S. enterica* subsp. *salamae* strains harbouring virulence genes underwent partial *stn* gene sequencing. Sequencing was selectively applied to the *stn* gene of *S. salamae* isolates due to its unexpected prevalence and limited molecular characterization in broiler chickens compared with commonly reported serovars. Amplicons were purified using a QIAquick purification kit (Qiagen, Germany). Afterwards, sequencing was performed on an ABI 3500 Genetic Analyzer (Applied Biosystems, USA) using the Big Dye Terminator V3.1 kit. The partial *S. enterica* subsp. *salamae stn* gene sequences retrieved in this study from broiler chickens were deposited in GenBank under the following accession numbers: PV855210 and PV816251. The obtained sequences were aligned against *stn* gene partial sequences of *Salmonella* strains isolated from humans, cows, and birds available on GenBank via ClustalW multiple alignment using BioEdit software version 7.0.9. A phylogenetic tree was constructed via the maximum-likelihood method with 500 replicates of the bootstrap consensus tree using MEGA 7 software (Fig. 1).

### Statistical analysis

Statistical analysis was conducted using chi-square tests, with Cramer’s V employed to assess the strength of association (Cramér 1946). The thresholds for interpreting Cramer’s V were as follows: Values ≤ 0.2 indicated a weak association, > 0.2 to ≤ 0.6 indicated a moderate association, and values > 0.6 indicated a strong association. To illustrate the distribution patterns of virulence and antimicrobial resistance genes among *Salmonella* isolates, a stacked proportional bar plot was generated using the ‘*tidyverse*’ (Wickham et al. 2019) and ‘*ggplot2*’ packages (Wickham et al. 2025). Pairwise gene-gene similarity was determined by calculating the Simple Matching Coefficient (SMC) based on binary presence/absence data. The SMC values ranged from 0 (completely dissimilar) to 1 (identical) and were visualized as annotated heatmaps using the ‘corrplot’ package (Wei and Simko 2024). Hierarchical clustering was performed on the binary presence/absence matrix of virulence and resistance genes, using the Jaccard distance metric, which measures the dissimilarity between two samples as the proportion of non-shared features to the total number of unique features present in either sample. The Jaccard distance ranges from 0 (identical profiles) to 1 (completely dissimilar profiles). The clustering pattern was conducted using the unweighted pair group method with arithmetic mean (UPGMA), via the ‘*vegan’* (Oksanen et al. 2001) and *‘cluster’* (Maechler et al. 1999) packages for Jaccard distance calculation. All visualizations were generated using R software (version 4.4.3). A *p*-value < 0.05 was considered statistically significant.

## Results

### Prevalence of *Salmonella* in broiler chickens

Out of 240 examined broiler chickens collected from 22 farms, *Salmonella* was detected in 19 birds (7.9%). The prevalence of *Salmonella* varied significantly by month (*p* = 0.030, Cramer’s *V* = 0.21), with the highest prevalence observed in April (20.0%). Significant difference was also shown among governorates (*p* = 0.002, Cramer’s *V* = 0.31), with Qalyubia reporting the highest infection rate (23.3%). However, no significant variation was found between the Lower and Middle Egypt regions (*p* = 0.811). Regarding flock characteristics, breed and age exhibited no significant association with *Salmonella* prevalence. On the other hand, ventilation type showed a significant association (*p* = 0.005, Cramer’s *V* = 0.21), with the highest detection rate in semi-closed systems (24.0%). Bird density also revealed a significant correlation (*p* = 0.015, Cramer’s *V* = 0.17), as farms with fewer than 10,000 birds had a higher prevalence (16.7%) compared to more densely populated farms (6.0%). Housing type (cages verus litter) and the number of antibiotic classes used (≤ 2 versus ≥ 3) were not significantly associated with *Salmonella* detection (*p* > 0.05 for both), as shown in Table 3.

**Table 3:** Association between various factors and *Salmonella* prevalence in broiler chickens in Egypt

Variables	Categories	+/total birds (%)	Cramer’s V	*P*-value
	February	9/90 (10.0)		
Month	March	1/60 (1.7)		
	April	6/30 (20.0)	**0.21**	**0.030**
	May	1/30 (3.3)		
	June	2/30 (6.7)		
Governorate	Qalyubia	7/30 (23.3)		
	Fayoum	6/30 (20.0)		
	Minya	2/30 (6.7)		
	Sharqia	2/30 (6.7)	**0.31**	**0.002**
	Bani Sweif	1/30 (3.3)		
	Gharbia	1/30 (3.3)		
	Beheira	0/30 (0.0)		
	Giza	0/30 (0.0)		
Location	Lower Egypt	10/120 (8.3)	0.02	0.811
	Middle Egypt	9/120 (7.5)		
Breed	Ross	6/65 (9.2)	0.05	0.960
	Cobb	11/140 (7.9)		
	Ivan-48	2/30 (6.7)		
	Sasso	0/5 (0.0)		
Age	Week 1	0/5 (0.0)		
	Week 2	1/10 (10.0)		
	Week 3	5/35 (14.3)		
	Week 4	7/60 (11.7)	0.15	0.176
	Week 5	6/130 (4.6)		
Ventilation	Semi-closed	6/25 (24.0)		
	Open	7/90 (7.8)	**0.21**	**0.005**
	Closed	6/125 (4.8)		
Housing system	Litter	16/200 (8.0)	0.01	1.000
	Cages	3/40 (7.5)		
Bird density	< 10,000	10/60 (16.7)	**0.17**	**0.015**
	> 10,000	9/150 (6.0)		
Antibiotic classes	≤ 2	9/115 (7.8)	0.03	0.675
	≥ 3	5/80 (6.3)		

+/total birds (%) indicates the number and percentage of birds that tested positive for *Salmonella* out of the total sampled within each category. Statistical significance was determined using the chi-square test with Cramer’s *V* as a measure of effect size. A score of ≤ 0.2 indicates weak association, > 0.2 to ≤ 0.6 shows moderate association, and > 0.6 denotes strong association. Bolded *p*-values reveal statistically significant associations (*p* < 0.05)

### Serotyping of *Salmonella enterica* isolates

Serotyping of the 19 *Salmonella* isolates identified *Salmonella* Enteritidis as the most prevalent serotype, accounting for 63.2% (12/19). This was followed by *Salmonella enterica* subsp. *salamae*, which showed a prevalence of 31.6% (6/19), and *Salmonella* Infantis, detected in a single isolate, representing 5.3% (1/19).

### Antimicrobial resistance profile of obtained *Salmonella* isolates

The antimicrobial susceptibility testing revealed cefpodoxime as the antibiotic with the highest resistance rate among the isolates at 89.5%, followed by nalidixic acid (73.7%), tetracycline (57.9%), cefoxitin (47.4%), nitrofurantoin (42.1%), and ceftazidime, azithromycin, and chloramphenicol (36.8%). Resistance to trimethoprim/sulfamethoxazole and ampicillin was recorded at 31.6%. Conversely, lower resistance rates were noted for ciprofloxacin (15.8%), cefotaxime (10.5%), aztreonam, ertapenem, and gentamicin (5.3% each). Notably, all isolates exhibited susceptibility to cefepime, meropenem, amikacin, and fosfomycin, as shown in Table 4. Multidrug resistance (MDR) to three or more antimicrobial classes was detected in 14 (73.7%) out of 19 isolates, with eight *S*. Enteritidis, five *S. enterica* subsp. *salamae*, and one *S*. Infantis being MDR. Furthermore, nine MDR isolates, comprising four *S*. Enteritidis, four *S. enterica* subsp. *salamae*, and one *S*. Infantis, displayed a MAR index greater than 0.2. The highest MAR index values were noted at 0.57 and 0.52 in one MDR *S*. Enteritidis and one MDR *S. enterica* subsp. *salamae*, respectively (Table 5).

**Table 4:** Antimicrobial resistance profile of *Salmonella* strains obtained from broiler chickens

Antimicrobial agent	*S*. Enteritidis (*n* = 12)	*S. enterica *subsp. *salamae* (*n* = 6)	*S*. Infantis (*n* = 1)	Total (*n* = 19)
Ceftazidime	3	4	0	7 (36.8%)
Cefotaxime	2	0	0	2 (10.5%)
Cefpodoxime	11	5	1	17 (89.5%)
Cefoxitin	4	4	1	9 (47.4%)
Cefoperazone	2	2	1	5 (26.3%)
Cefepime	0	0	0	0
Aztreonam	1	0	0	1 (5.3%)
Ertapenem	1	0	0	1 (5.3%)
Meropenem	0	0	0	0
Trimethoprim/sulfamethoxazole	2	4	0	6 (31.6%)
Nalidixic acid	8	5	1	14 (73.7%)
Ampicillin	4	1	1	6 (31.6%)
Gentamicin	1	0	0	1 (5.3%)
Amikacin	0	0	0	0
Azithromycin	4	2	1	7 (36.8%)
Nitrofurantoin	3	4	1	8 (42.1%)
Chloramphenicol	6	1	0	7 (36.8%)
Ciprofloxacin	1	1	1	3 (15.8%)
Fosfomycin	0	0	0	0
Tetracycline	5	5	1	11 (57.9%)
Doxycycline	2	1	1	4 (21.1%)

**Table 5:** Antimicrobial resistance pattern and MAR index of multidrug-resistant *Salmonella* strains

Isolate code	*Salmonella* isolate	Resistance pattern	MAR index
Fb9	*S*. Enteritidis	CTX-CPD-CX-NA-AZM-CIP	0.28
Fb10	*S*. Enteritidis	CPD-NA-C	0.14
Fb13	*S*. Enteritidis	CAZ-CTX-CPD-AT-NA-AMP-GEN-NIT-C-TE	0.47
Fb25	*S*. Enteritidis	CPD-NA-AZM-C	0.19
SH25	*S*. Enteritidis	CPD-CX-CFZ-ET-SXT-NA-AMP-AZM-NIT-C-TE-DO	0.57
MB30	*S*. Enteritidis	CPD-AMP-TE	0.14
Qb16	*S*. Enteritidis	CAZ-CPD-CX-SXT-NA-NIT-TE	0.33
Qb14	*S*. Enteritidis	CAZ-CPD-CX-CFZ-NA-AMP-C-TE-DO	0.42
Fb16	*S. enterica* subsp. *salamae*	CPD-CX-CFZ-SXT-NA-AMP-NIT-C-CIP-TE	0.47
Bb14	*S. enterica* subsp. *salamae*	CAZ-CPD-NA-TE	0.19
Mb14	*S. enterica* subsp. *salamae*	CAZ-CPD-CX-SXT-NA-NIT-TE	0.33
Qb21	*S. enterica* subsp. *salamae*	CAZ-CPD-CX-CFZ-SXT-NA-AMP-AZM-NIT-TE-DO	0.52
Qb27	*S. enterica* subsp. *salamae*	CAZ-CPD-CX-SXT-NA-NIT-TE	0.33
Qb9	*S*. Infantis	CPD-CX-CFZ-NA-AMP-AZM-NIT-CIP-TE-DO	0.47

Ampicillin (AMP), cefotaxime (CTX), ceftazidime (CAZ), cefpodoxime (CPD), cefoxitin (CX), cefoperazone (CFZ), aztreonam (AT), gentamicin (GEN), azithromycin (AZM), tetracycline (TE), doxycycline (DO), ciprofloxacin (CIP), nalidixic acid (NA), trimethoprim-sulfamethoxazole (SXT), chloramphenicol (C), nitrofurantoin (NIT), and ertapenem (ET)

### Distribution of antimicrobial resistance genes among *Salmonella* strains

**Fig. 2:**
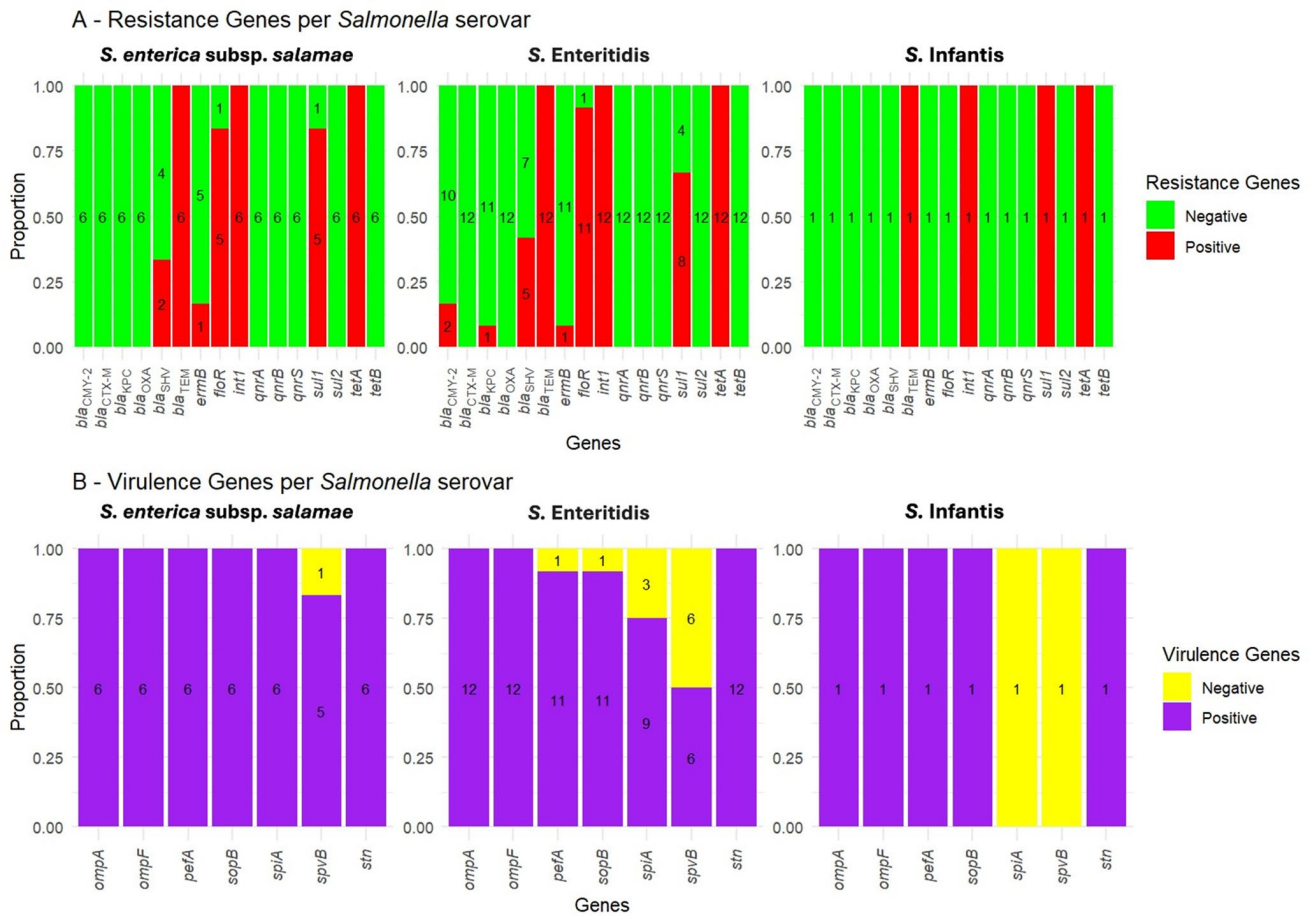
Proportional distribution of (**A**) antimicrobial resistance and (**B**) virulence genes among *Salmonella* species. Each bar represents the proportion of positive and negative detections per gene across isolates. Genes are plotted on the x-axis, with colours representing purple/red (positive) and yellow/green (negative). Faceting was used to distinguish between different *Salmonella* spp

The proportional distribution of antimicrobial resistance genes among *Salmonella* spp. was illustrated in Fig. 2A. All *Salmonella* isolates harboured *tetA*, *int1*, and *bla*_TEM_. In contrast, the *tetB*, *sul2*, *qnrA*, *qnrB*, *qnrS*,* bla*_CTX−M_, *bla*_OXA_, *bla*_NDM_, *bla*_OXA−48_, and *bla*_VIM_ genes were not amplified in all *Salmonella* isolates. The *sul1* gene was detected in 8 *S*. Enteritidis, 5 *S. enterica* subsp. *salamae*, and one *S*. Infantis isolate. Additionally, *bla*_SHV_ and *floR* were recognized in 5 and 11 strains of *S*. Enteritidis, respectively, as well as in 2 and 5 isolates of *S. enterica* subsp. *salamae*. The *ermB* gene was found in one isolate from both *S*. Enteritidis and *S. enterica* subsp. *salamae*. Comparatively, two *S*. Enteritidis isolates possessed the *bla*_CYM−2_ gene, while one isolate carried *bla*_KPC_.

### Distribution of virulence genes among *Salmonella* strains

All 12 *S*. Enteritidis isolates tested positive for the *stn*, *ompA*, and *ompF* genes. Additionally, 11 of these isolates were found to carry *pefA* and *sopB*, while *spvB* and *spiA* were present in six and nine strains, respectively. Interestingly, all six *S. enterica* subsp. *salamae* isolates tested positive for *stn*, *spiA*, *sopB*, *pefA*, *ompA*, and *ompF*, with *spvB* detected in five. Regarding the single *S*. Infantis isolate, it displayed the presence of all tested virulence genes except *spiA* and *spvB*, as shown in Fig. 2B.

### Correlation between antimicrobial resistance genes and virulence determinants across *Salmonella* isolates

**Fig. 3:**
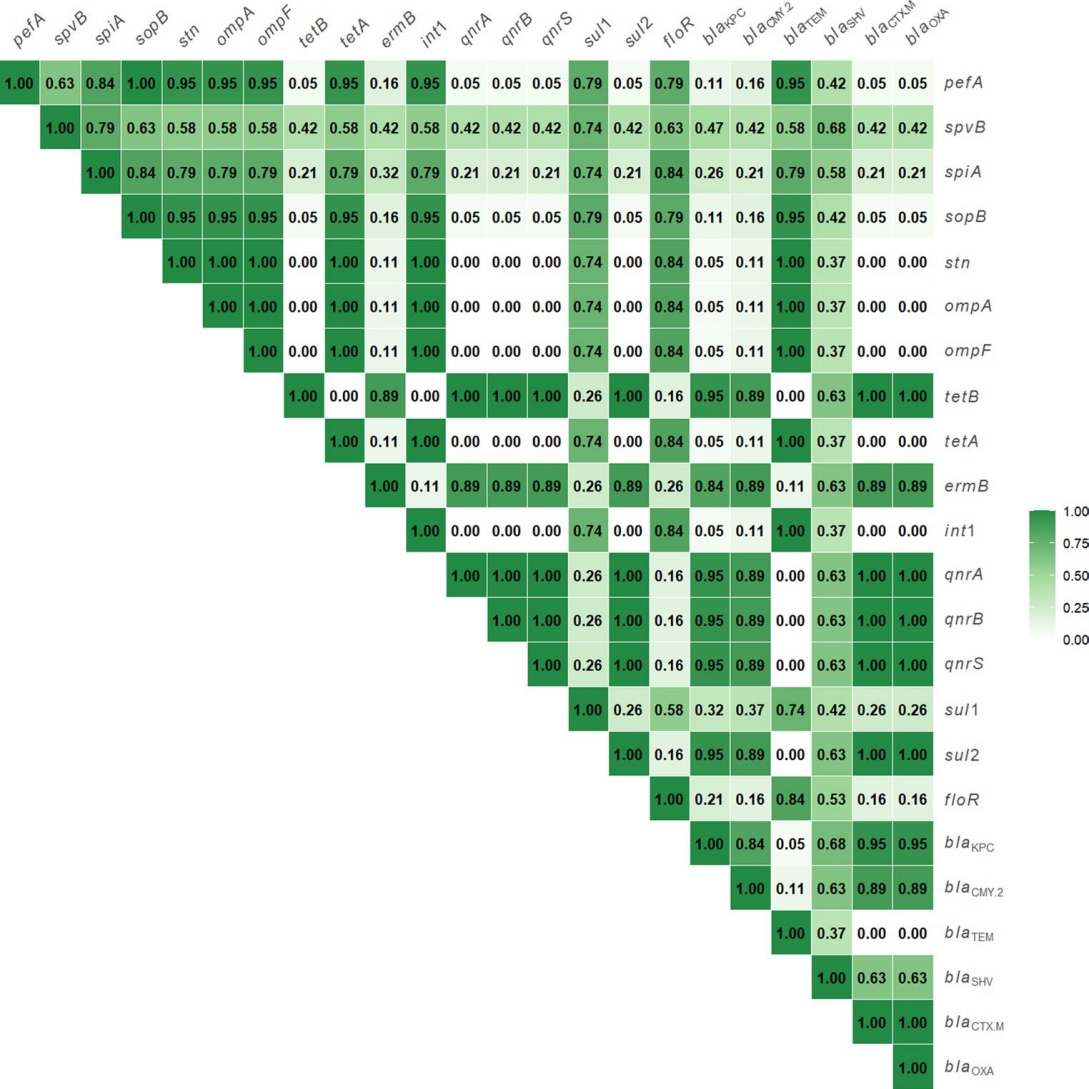
Heatmap of pairwise gene-gene similarities calculated using the Simple Matching Coefficient (SMC). Colour intensity denotes SMC values, with exact coefficients shown in each cell. Darker shades (values closer to 1) indicate stronger similarity in gene presence/absence patterns among isolates, whereas lighter shades (values closer to 0) indicate weaker similarity

Figure 3 plots the SMC values, which ranged from 0.00 to 1.00. Perfect similarity (SMC = 1.00) was observed among *stn*, *ompA*, *ompF*, *tetA*, *Int1*, and *bla*_*TEM*_, which were consistently identified in 100% of isolates. An identical pattern (SMC = 1.00) was also noted between *pefA* and *sopB*. High similarity (SMC > 0.80) was observed between *spiA* and each of *pefA*, *sopB*, and *floR*. Likewise, *ermB* demonstrated high similarity (SMC > 0.80) with both *bla*_*KPC*_ and *bla*_*CMY−2*_. Moreover, a strong similarity (SMC = 0.84) was detected between *bla*_*KPC*_ and *bla*_*CMY−2*_.

### Hierarchical clustering of *Salmonella* isolates

**Fig. 4:**
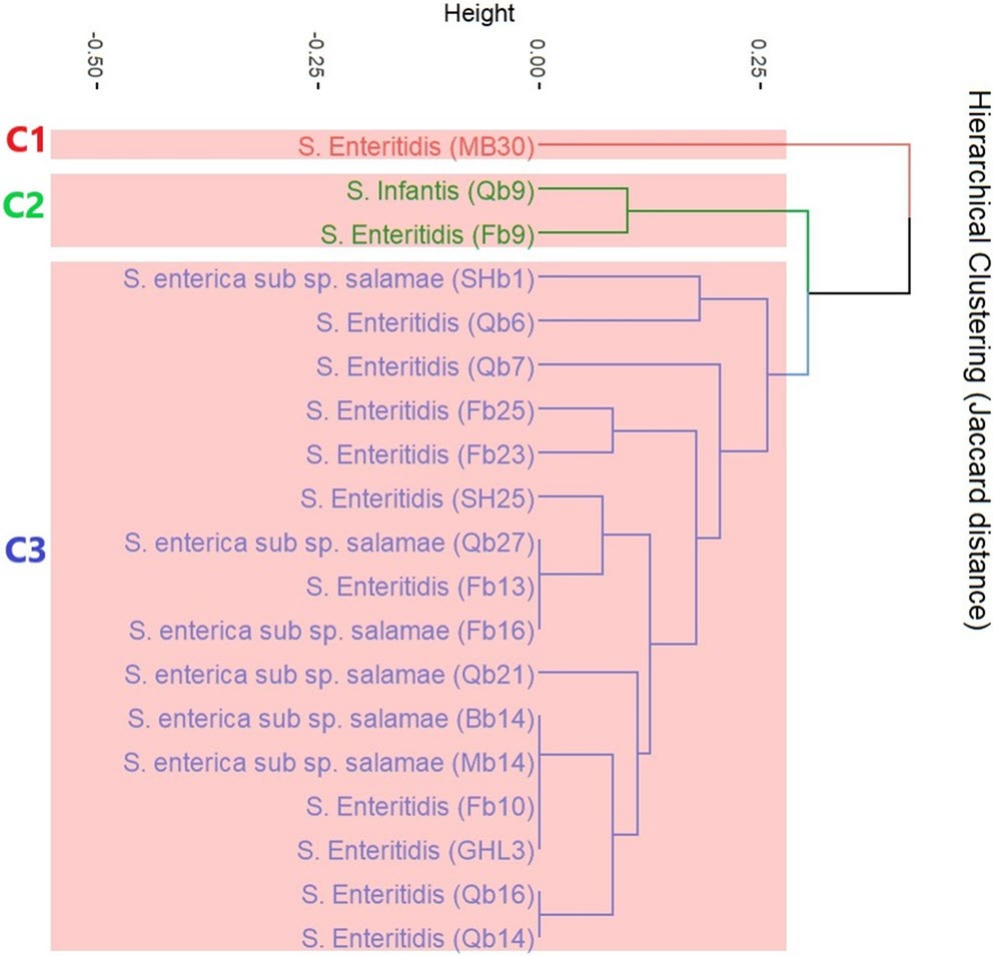
Hierarchical clustering of *Salmonella* isolates based on Jaccard distance was calculated from the presence/absence of virulence and antimicrobial resistance genes. The dendrogram was created using the unweighted pair-group method with arithmetic mean (UPGMA). Clusters (C1 - C3) were identified at a Jaccard distance threshold of 0.25, with red boxes indicating cluster boundaries. Isolate labels correspond to *Salmonella* species and code, while branch lengths reflect genetic dissimilarity (0 = identical profiles, 1 = completely different)

The hierarchical clustering pattern of *Salmonella* isolates (Fig. 4) revealed Jaccard distance values ranging from 0.00 to 0.40 among the isolates, indicating that some strains shared identical genetic profiles, whereas others exhibited substantial divergence in their genetic composition. Hierarchical clustering based on the Jaccard distance grouped the isolates into three main clusters, with closely related profiles (Jaccard distance ≤ 0.25) forming distinct subgroups. Cluster C1 comprised *S.* Enteritidis (MB30), which harboured the fewest genes (7/26), including three virulence (*stn*, *ompA*, and *ompF*) and four resistance genes (*tetA*, *Int1*, *floR*, and *bla*_*TEM*_). Cluster C2 encompassed two *Salmonella* isolates, *S.* Infantis (Qb9) and *S.* Enteritidis (Fb9), both exhibiting an identical profile with nine shared genes (*pefA*, *sopB*, *stn*, *ompA*, *ompF*, *tetA*, *Int1*, *sul1*, and *bla*_*TEM*_). The main distinction between them was the presence of *bla*_CMY−2_, exclusively detected in *S*. Enteritidis (Fb9) and absent in *S*. Infantis (Qb9). Notably, cluster C3 comprised all *S. enterica* subsp. *salamae* isolates alongside the remaining *S.* Enteritidis isolates. This cluster had an average of 12 genes per isolate (ranging from 9 to 15). All isolates in C3 shared eight genes (*pefA*, *sopB*, *stn*, *ompA*, *ompF*, *tetA*, *Int1*, and *bla*_*TEM*_). As well, most isolates carried an additional four genes (*spvB*, *spiA*, *sul1*, and *floR*), while approximately half had the *bla*_*SHV*_ gene.

## Discussion

In the present study, *Salmonella* spp. were detected in 19 broiler chickens with a prevalence of 7.9%. Across various continents, the studies highlight significant differences in the prevalence of *Salmonella* in poultry. In Africa, the overall pooled prevalence was reported as 14.4% (Kabeta et al. 2024). Within the European Union in 2024, *Salmonella*-positive samples from broiler carcasses reached 18.7% (EFSA and ECDC 2025). In the Americas, the pooled prevalence of non-typhoidal *Salmonella* in poultry samples stood at 17.9% (Diaz et al. 2022). Regionally, within individual countries, China reported the highest prevalence of *Salmonella* in raw poultry meat in Shaanxi (44.3%), followed by Henan (35.3%), Sichuan (35.0%), and Beijing (31.1%) (Sun et al. 2021). In Ethiopia, the highest pooled prevalence in poultry farms was observed in Western Ethiopia (23.18%), with lower rates in Central Ethiopia (11.98%) and Southern Ethiopia (10.3%) (Basazinew et al. 2025). Meanwhile, in the United States, the prevalence of *Salmonella* in broiler carcasses varied by region, with the Atlantic region showing an average incidence of 6.04% and the South Central region averaging 4.29% (Siceloff et al. 2022). This deviation might be due to differences in the methodology used during sample collection, the health status of the birds tested, environmental conditions, variations in poultry densities, farming practices, geographical locations, and biosecurity measures adopted (Basazinew et al. 2025).

When examining chicken breeds, a high prevalence of *Salmonella* was observed in Ross and Cobb breeds, whereas no instances of *Salmonella* infection were recorded in Sasso chickens. The elevated prevalence in Ross and Cobb breeds might be attributed to their rapid growth rate and high meat production capacity—characteristics that make them more susceptible to intensive production methods and inadequate biosecurity measures (Basazinew et al. 2025). The prevalence of *Salmonella* across housing systems revealed similar rates between the cage and deep litter systems. These findings were consistent with a study conducted in Ethiopia (Basazinew et al. 2025), which reported *Salmonella* prevalence in deep litter systems to be slightly higher by 0.55% compared to cage systems. In addition, this study identified a significant association between *Salmonella* prevalence and stocking density, with a higher percentage in flocks of fewer than 10,000 birds. This disparity might be due to enhanced biosecurity measures implemented by farms with higher stocking densities in Egypt, effectively reducing the risk of *Salmonella* infection. Notably, Qalyubiya recorded the highest prevalence of *Salmonella* at 23.3%, with statistical significance in this study. This governorate plays a central role in Egypt’s broiler chicken production, with production volumes averaging roughly 59.866 million chickens annually, according to Elbateh and Elhabbaq (2024). Variations in climate are likely to directly impact the distribution and diversity of *Salmonella* serovars. In Brazil’s tropical regions, which experience only rainy and dry seasons—with rainfall concentrated in a few months and minimal temperature fluctuations (average > 25 °C)—the prevalence of *Salmonella* remains uncertain (Calle et al. 2021). Temperatures exceeding 25 °C increase the likelihood of *Salmonella* spp. survival (Hwang et al. 2020). Similarly, the high humidity during the rainy season may hinder the detection of *Salmonella* spp. in chicken feces in tropical areas of Brazil by promoting microbial fermentation (Miranda et al. 2026). In Mexico, states with hot-humid climates report the highest rates of non-typhoidal salmonellosis. Climatic factors such as high temperatures and increased rainfall encourage the proliferation of *Salmonella* spp., potentially enhancing bacterial replication and transmission to surface water and food crops, thereby escalating risks of infection (Flores Monter et al. 2021). However, this study observed the highest *Salmonella* prevalence was in April (Spring season) at 20%, revealing a significant correlation.

Serotyping serves as a fundamental biomarker for examining the epidemiological status of *Salmonella* infections and is commonly employed to trace contamination sources during outbreaks (Bell et al. 2016). Interestingly, this study revealed an unexpected distribution of serovars, with *S. enterica* subsp. *salamae* being identified in 31.6% of broilers, trailing behind *S*. Enteritidis (63.2%) and followed by *S*. Infantis (5.3%). These findings diverge from results in other studies investigating *Salmonella* prevalence in broiler chickens across various countries, with *S*. Enteritidis and *S*. Typhimurium consistently identified as the dominant serovars (Algammal et al. 2023; Tohamy et al. 2022; Siddiky et al. 2021; Williams et al. 2025). *S. enterica* has received significant attention from researchers, though much of this focus has been directed towards its subspecies *enterica*, particularly the serovars Enteritidis and Typhimurium. However, there is a notable lack of information regarding other subspecies of *S. enterica*, including *S. enterica* subsp. *salamae* (Lamas et al. 2018). *S. salamae* has been identified in various animal hosts, such as pet and wild reptiles (Marin et al. 2021; Mlangeni et al. 2024), lizards (Zając et al. 2021), free-living turtles (Casalino et al. 2021), and snakes (Dec et al. 2022). Beyond reptiles, this subspecies has also been detected in fish (Baniga et al. 2019), livestock and associated products (Fatima et al. 2023; Wilson et al. 2025), as well as environmental sources (Ofori et al. 2023). In the field of poultry research, *S. salamae* has been isolated from poultry meat in South Africa (Ndobeni et al. 2023) and broiler chickens in Egypt (Shalaby et al. 2022). It has also been detected in turkeys (Gouda et al. 2020) and within poultry houses (Lamas et al. 2021). From a public health perspective, few clinical reports documented *S. enterica* subsp. *salamae* infections in humans. For instance, *S. enterica* subsp. II serovar 4,5,12:a:- has been identified as a potential cause of gastroenteritis in humans where whole genome sequencing has revealed that isolates of this serovar are closely related to subspecies *salamae* strains (Yan et al. 2022). Besides, *S*. *salamae* has shown the ability to cause extra-intestinal infections in humans. It was identified in a pediatric case of infective endocarditis, and surveillance data show isolates from extraintestinal sites (e.g., urine) attributable to subspecies II organisms, as noted by Dhayhi et al. (2021). Although previous reports indicated a low incidence of *S. enterica* subsp. *salamae*, our research findings reveal a higher prevalence, raising concerns about its transmission pathway and suggesting that the consumption of poultry meat may facilitate the spread of these strains, similar to documented cases of human infection associated with reptile meat consumption (López-Quintana et al. 2015).

Antibiotics are the magic bullets for tackling life-threatening infections. However, the misuse and overuse of these vital medications in poultry farms are driving the rapid progression of multidrug-resistant bacteria (Abreu et al. 2023). In this study, a high resistance was observed towards cefpodoxime, nalidixic acid, and tetracycline, while moderate resistance was reported for cefoxitin, nitrofurantoin, ceftazidime, azithromycin, chloramphenicol, trimethoprim/sulfamethoxazole, and ampicillin. Similarly, Ammar et al. (2019) found notable resistance to nalidixic acid (93%), sulfamethoxazole/trimethoprim (40%), and ampicillin (37%) among *Salmonella* serovars isolated from broiler chickens in Egypt. The resistance to nalidixic acid closely aligned with the findings of Siddiky et al. (2021), who documented 60% to 86.7% resistance to nalidixic acid in *Salmonella* strains isolated from chickens in Bangladesh. The high prevalence of resistance to tetracycline and nalidixic acid observed in this study suggests frequent usage of these antibiotics in Egypt’s poultry farming system. The plasmid-mediated nature of tetracycline resistance and the chromosomal stability of quinolone resistance mechanisms facilitate the persistence of these resistant *Salmonella* strains within broiler production systems. In the present study, the high resistance of *Salmonella* isolates to cefpodoxime may be attributed to the widespread dissemination of β-Lactamase-producing strains in poultry production systems. The use of cephalosporins or other β-lactam antibiotics in poultry production may exert selective pressure, leading to cross-resistance to cefpodoxime. Noteworthy, this study revealed that the majority of *S. enterica* subsp. *salamae* isolates exhibited resistance to ceftazidime, cefpodoxime, cefoxitin, trimethoprim/sulfamethoxazole, nalidixic acid, nitrofurantoin, and tetracycline. Gouda et al. (2020) found that *S. salamae* isolated from turkey were resistant to ampicillin, ceftazidime, ciprofloxacin, and chloramphenicol. On the other hand, all *Salmonella* isolates, including subspecies *salamae* in this study, exhibited sensitivity to carbapenem. This was consistent with findings of Gouda et al. (2020), who reported that two *S. enterica* subsp. *salamae* isolates recovered from turkeys were sensitive to imipenem. The lack of resistance to carbapenem could be linked to the absence of its use for prevention or treatment in commercial chicken farms in Egypt (Elkenany et al. 2019). Multidrug-resistant (MDR) *Salmonella* is predominantly associated with subspecies *enterica* serotypes, specifically *S*. Typhimurium and *S*. Enteritidis, but there is little knowledge regarding multidrug resistance in non-*enterica* subspecies (Lamas et al. 2018). Unfortunately, the current study revealed that 73.7% of the isolates exhibited MDR. This result was compatible with those obtained by Elkenany et al. (2019), who reported MDR in 76.7% of isolates. The high proportion of MDR *Salmonella* isolates observed in broiler chickens likely reflects the combined effects of extensive antimicrobial use and the efficient dissemination of resistance determinants within poultry production systems. The MAR index is recognized as a cost-effective and reliable method to track the source of bacteria (Mir et al. 2022). In an astonishing way, this study identified five MDR *S. enterica* subsp. *salamae* isolates, four of which had MAR indices exceeding 0.2. MAR indices larger than 0.2 suggest that these isolates originated from high-risk contamination sources where antibiotics are frequently used and/or in large amounts (Mthembu et al. 2019). These findings underscore the growing significance of non-*enterica* subspecies as a reservoir for antimicrobial resistance within poultry production systems, posing potential risks for food safety and public health.

The current investigation demonstrated that all *S. enterica* subsp. *salamae* strains carried the *bla*_TEM_ gene, but no bands indicating the *bla*_CTX−M_ or *bla*_OXA_ genes were identified. Similar to this, Gouda et al. (2020) recognized *bla*_TEM_ in *S. enterica* subsp. *salamae* isolates obtained from turkeys, whereas *bla*_OXA_ was undetectable. Interestingly, the *tetA* gene was identified across all *Salmonella* isolates in this study, including *S. enterica* subsp. *salamae*, despite tetracycline resistance being observed in 57.9% of isolates. This suggests that some of the antimicrobial resistance genes are silent in bacteria in vitro; nevertheless, these silent genes have the potential to be transmitted to other bacteria or turn on in vivo, particularly when subjected to antimicrobial pressure (El-Sharkawy et al. 2017). Furthermore, class 1 integron (*intl1*) was detected in all *Salmonella* strains analyzed in this study, including those belonging to *S. salamae*. This result contrasts report of Alam et al. (2020), who observed this gene in 20% of *Salmonella* strains. Regarding sulfonamide resistance genes, only *sul1* was detected in five *S. enterica* subsp. *salamae*. Arkali and Çetinkaya (2020) previously reported the presence of the *sul1* gene in 58% of *Salmonella* isolates retrieved from chickens in eastern Turkey. Additionally, *floR* was identified in five *S. enterica* subsp. *salamae* isolates in this work. In comparison, a previous study detected the *floR* gene in two *S. enterica* subsp. *salamae* isolates (Abd El-Tawab et al. 2020). The phenotypic and genotypic antimicrobial resistance profiles point out that broiler chickens could be a potential reservoir for MDR *S. enterica* subsp. *salamae*, which harbours antimicrobial resistance genes. This emphazises the need for conducting further research on non-*enterica* subspecies of *Salmonella*.

While previous research indicated that *S. enterica* subsp. *salamae* tends to exhibit lower pathogenicity compared to *S. enterica* serovars (Lamas et al. 2018), findings from this study revealed a contrasting outcome. All six *S. salamae* isolates in the study tested positive for the *stn*, *ompA*, and *ompF* genes. Previous studies have found that all *Salmonella* strains had *ompA* (Orabi et al. 2022), while the *ompF* gene was present in 45.45% (Elsayed et al. 2024). The *Salmonella* enterotoxin (*stn*) gene was demonstrated as a suitable PCR target for detection, serotyping, and virulence analysis of *Salmonella* strains (Fu et al. 2025). Consistent with our findings, *stn* was identified in all *Salmonella* isolates in study carried out by Shen et al. (2023). The *pefA* and *sopB* genes were detected in six *S*. *enterica* subsp. *salamae *in the current study; Lozano-Villegas et al. (2023) recognized *pefA* and *sopB* in 74.4% and 100% of poultry *Salmonella* isolates, respectively. This study demonstrated that all six *S. enterica* subsp. *salamae* isolates possessed the *spiA* gene, consistent with findings by Lozano-Villegas et al. (2023). The prevalence of *spiA* in broilers is particularly significant as it enhances the ability of *Salmonella* serotypes to form biofilms, a trait known to prolong their survival in poultry farming environments (Chen et al. 2025). Therefore, *Salmonella* strains harbouring this gene could persist on farms and contaminate meat and eggs, posing a public health risk through foodborne transmission to humans (Lozano-Villegas et al. 2023). Additionally, Lozano-Villegas et al. (2023) reported a 71.8% prevalence of *spvB* in poultry isolates, aligning closely with the current study’s findings (five *S. salamae* isolates had *spvB*). This gene is relevant because *spv* genes are highly associated with strains that cause enteritis, or non-typhoidal disseminated infections in humans (Zhao et al. 2025). These results clarified that *S. enterica* subsp. *salamae* isolates have a broad virulence profile. In the meantime, the hierarchical clustering of *Salmonella* isolates revealed all *S. enterica* subsp. *salamae* isolates fall within the same cluster with *S.* Enteritidis strains obtained in this study, reflecting the potential threat of *salamae* strains.

Remarkably, in this research, the strongest correlation was exhibited among *stn*, *ompA*, *ompF*, *tetA*, *Int1*, and *bla*_*TEM*_, all of which were consistently found in 100% of isolates. Furthermore, *spiA* shared a high similarity with *pefA*, *sopB*, and *floR*. The significant association between antibiotic resistance and virulence-associated genes in *Salmonella* strains is attributed to genetic determinants for these traits often being located within the same mobile genetic elements (MGEs) (Han et al. 2025). Because the *Salmonella stn* gene is a key marker for recognizing *Salmonella* strains (Fu et al. 2025) and refers to their public health significance, being responsible for gastroenteritis and diarrhoea (Petano-Duque et al. 2023), partial sequencing of the *stn* gene was carried out on two MDR isolates belonging to *S. enterica* subsp. *salamae*. Afterwards, a phylogenetic tree was constructed to illustrate the genetic relationships between partial *stn* gene sequences of *S. enterica* subsp. *salamae* obtained in this study and those available on GenBank records. The analysis revealed that the *S. salamae stn* gene sequence retrieved from broiler chicken in this work (PV816251) clustered within the same clade with the *S. enterica* strain isolated from a human in Egypt (LC227779). Furthermore, the tree demonstrated a close genetic relationship between another *S. enterica* subsp. *salamae* strain isolated from broiler chicken (PV855210) in this investigation, strains derived from wild migratory birds in Egypt (MZ868594), and human stool samples in Iraq (MK894582). These findings underscore the zoonotic potential of *S. enterica* subsp. *salamae* and their public health significance.

## Conclusion

This study highlighted the prevalence, antibiotic resistance, and virulence of *S. enterica* subsp. *salamae* in broiler chickens in Egypt. The identification of virulent and MDR *S. enterica* subsp. *salamae* strains in broiler chickens alongside *S*. Enteritidis, a main *S. enterica* serovar associated with human salmonellosis, raises a potential public health concern. Knowledge concerning non-*enterica* subspecies remains limited, so this study opens up avenues for researchers to explore their virulence and potential for human colonization more thoroughly. Whole genome sequencing should be the preferred option to determine the genetic variations between *S. enterica* subspecies, allowing us to better understand their pathogenicity. Ultimately, active surveillance and data sharing among the human, animal, and environmental sectors under the umbrella of the One Health strategy are essential when investigating emerging *Salmonella* spp., antimicrobial resistance, and virulence factors to reduce economic losses and human health risks.

## Data Availability

All data generated or analyzed during this study are included in this published article. The partial *Salmonella enterica* subsp. *salamae stn* gene sequences generated in this study from broiler chickens were deposited in GenBank under the following accession numbers: PV855210 and PV816251.
